# Immediate Temporal Information Modulates the Target Identification in the Attentional Blink

**DOI:** 10.3390/brainsci12020278

**Published:** 2022-02-17

**Authors:** Fangshu Yao, Bin Zhou, Yiyun Zhuang, Xiaochun Wang

**Affiliations:** 1 School of Psychology, Shanghai University of Sport, Shanghai 200438, China; yaofangshu@sus.edu.cn (F.Y.); 19600310@sus.edu.cn (Y.Z.); 2 State Key Laboratory of Brain and Cognitive Science, Institute of Psychology, Chinese Academy of Sciences, Beijing 100101, China

**Keywords:** temporal uncertainty, attentional blink, interval cue, rhythmic cue, conscious processing

## Abstract

It has been shown that learned temporal information can be exploited to help facilitate the target identification in the attentional blink task. Here, we tested whether similar exploitation also worked on short-term temporal information, even when it did not reliably predict the target onset. In two experiments, we randomly manipulated either the interval between targets (T1 and T2; Experiment 1) or the temporal regularity of stimulus presentation (Experiment 2) in each trial. The results revealed evidence of effects of immediate temporal experience mainly on T2 performances but also occasionally on T1 performances. In general, the accuracy of T2 was enhanced when a longer inter-target interval was explicitly processed in the preceding trial (Experiment 1) or the temporal regularity, regardless of being explicitly or implicitly processed, was present in the stimulus stream, especially after T1 (Experiment 2). These results suggest that, under high temporal uncertainty, both interval and rhythmic cues can still be exploited to regulate the allocation of processing resources, thus, modulating the target identification in the attentional blink task, consistent with the view of flexible attentional allocation, and further highlighting the importance of the interplay between temporal processing and attentional control in the conscious visual perception.

## 1. Introduction

Although our visual system is capable of sampling inputs with high temporal resolution on the scale of tens of milliseconds [1], we still suffer severe temporal limitations when identifying successive items presented within about half a second. Such constraint in conscious report is assumed to be associated with the operation of attentional functions, and is demonstrated clearly in the attentional blink (AB) phenomenon [2,3], where the success in identifying the first target (T1) in a rapid serial visual presentation (RSVP) stream often comes at the expense of the second target (T2), in stark contrast to the high accuracy when only T2 is to be reported. The performance of judging T2 does not fully recover until the interval between targets goes beyond about 500 ms (referred to as lag5 under the convention of AB). This phenomenon likely arises due to limited central processing resources [4,5] or sub-optimal allocation of these resources on especially T2 processing when it shortly follows T1 [6,7,8]. When T2 appears immediately after T1, the T2 performance is sometimes spared, leading to the lag1 sparing, which is assumed to benefit from both targets being in the same attentional episode [9].

The AB effect, sometimes also the lag1 sparing, is modulated by various factors [10,11,12,13,14]. One factor, which has gained much interest in the past decade, is the temporal information that can be used to adjust the attentional allocation particularly between targets, which is most relevant to the AB effect. Indeed, the AB effect can be alleviated by manipulating the proportion of lag conditions within a block [15,16,17] or by pre-training with the to-be-probed lag rather than variable lags [18,19,20]. T2 performances at short lags can also be enhanced by symbolically cueing the exact time when T2 will appear [21,22,23]. In addition to such interval-based time cues, the temporal regularity of the RSVP stream can modulate the AB effect as well [24,25]. Entraining neural activities with rhythmic cues before the RSVP onset [26,27] or nesting additional rhythmic contexts in the RSVP stream [28,29] also has the potential to alleviate the AB effect. These observations suggest that information about temporal structures can be exploited to organize the allocation of attentional resources to multiple targets in the RSVP stream, thus, reducing the AB deficit.

Previous AB studies usually required observers to access interval information by either cognitive transformation [21,22,23] or statistical learning [15,16,17,18]. However, many daily situations often involve an unpredictable occurrence of stimuli and have high temporal uncertainty which cannot be resolved via explicit cueing or long-term learning. It has been shown that temporal information can be readily extracted on the single-trial level without semantic cues and modulates behaviors thereafter [30,31]. Can such T1-T2 interval, per se, be instantaneously processed as a time cue to facilitate the target identification in situations with high temporal uncertainty? No clear answer to this question has yet been achieved. Similarly in the rhythmic presentation of stimuli, temporal uncertainty can be mitigated by exploiting the rhythmicity of the sequence [32,33]. However, most AB studies employ RSVPs with uniform presentation rates; it remains largely unclarified how variant constituents of the serial presentation, such as different rates within a sequence, may modulate the AB effect. Given that both interval and rhythmic cues can effectively direct attentional resources to anticipated moments and, thus, reduce temporal uncertainty [32], answering the above questions will help illuminate the interaction between time processing and temporal control of resources, as well as AB mechanisms.

To elucidate the role played by both interval-based and rhythm-based temporal processing, we conducted two experiments to respectively investigate the effect of the inter-target interval between successive trials (Experiment 1) and of the temporal regularity before and after T1 within trials (Experiment 2). As we were more interested in the modulatory effect of temporal information on the attentional allocation between targets rather than processes within one attentional episode, our focus was on the AB effect. Furthermore, the interval-based and rhythm-based time cues usually act on different aspects of behaviors [34,35] and engage dissociated neural mechanisms [36,37]. Specifically, rhythm-based timing, compared with interval-based timing, operates more automatically and relies less on conscious processing [38,39]. If such dissociation also exists in the temporal control of attentional resources, we would observe that the interval cueing effect, relative to rhythmic cueing effect, is more dependent on the deliberate or conscious processing of time cues. We, therefore, further explored in both experiments whether actively processing and passively exposing to the temporal information would differently modulate the behavioral performance in the AB task.

## 2. Experiment 1: Effect of Interval Cues

### 2.1. Method

#### 2.1.1. Participants

Altogether, 44 healthy adults (eight males, 19.86 ± 1.03 years), randomly assigned into the “explicit-timing” and “implicit-timing” groups, voluntarily participated in Experiment 1. To obtain enough statistical power, the sample size was no less than the recommendation by a power analysis using G*Power 3.1.9.2 [40], given a conventional medium effect size (f = 0.25) [41] according to previous AB studies [17,25], an alpha level set at 0.05, and an estimated power of >0.8. We excluded data from participants whose overall T1 accuracies were lower than two standard deviations from the group mean, resulting in a final dataset from 21 participants in the explicit-timing group and 20 participants in the implicit-timing group. Participants in both groups were not informed of the aim of the experiment nor the presentation manipulation before the task. All participants were right-handed and had normal or corrected-to-normal vision. They all provided informed consent before and received reimbursement after the experiment. The procedure was in accord with the Declaration of Helsinki and further approved by the Ethics Committee in Shanghai University of Sport.

#### 2.1.2. Apparatus, Stimuli, and Procedure

Visual stimuli were presented on a 24-inch LED monitor (LT2423wC, Lenovo Inc., Beijing, China) connected to a PC running MATLAB R2016b (^®^MathWorks) and Psychophysics Toolbox extension Version 3 [42] in a quiet, dimly lit room. The refresh rate of the monitor was set to 60 Hz and the background was black (RGB: 0, 0, 0). The rapid serial visual presentation (RSVP) stream consisted of 16 items presented in Courier New Bold font, including all letters of the English alphabet except E and O, for their structural similarity to F and Q, respectively, and I and L, for their mutual resemblance. Items in each trial were randomly chosen without repetition, with distractors in white (RGB: 255, 255, 255) and target(s), one or two depending on the experimental condition, in green (RGB: 0, 255, 0). All stimuli were displayed at the center of the screen and subtended 1.5° of visual angle when viewed from a distance of 60 cm.

**Figure 1:**
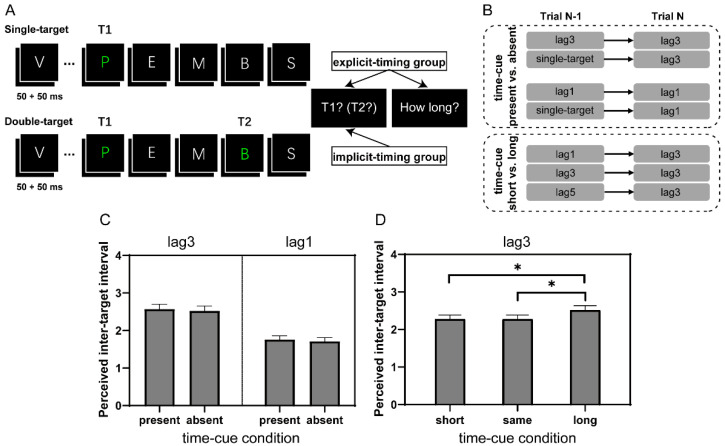
The experimental paradigm of the AB task and subjective rating scores of time perception in Experiment 1. (**A**) Schematic representation of a single-target or a double-target (lag3) trial in Experiment 1. (**B**) The inter-target interval was manipulated between successive trials. (**C**) Ratings of perceived inter-target interval in the time-cue present vs. absent comparison at lag3 and lag1, respectively. (**D**) The same as (**C**) in the time-cue short vs. long comparison at lag3. The vertical axis indicates “only see one target (1)”, “relatively short (2)”, “medium (3)”, and “relatively long (4)”. Error bars represent standard error. * *p* < 0.05. All multiple comparisons were Bonferroni corrected.

Each trial began with the presentation of a white central fixation cross for 1000 ms, followed by an RSVP stream in which items were presented for 50 ms each with another 50 ms blank interval in between, enabling a presentation rate of 10 Hz (Figure 1A). The first target (T1) was randomly selected as the fifth, sixth, or seventh item in the stream and the second target (T2), if present, was the first, third, or fifth item following T1, yielding conditions of lag1 (100 ms), lag3 (300 ms), or lag5 (500 ms) correspondingly. Upon the offset of the stream, participants were prompted to report the identity of all targets they thought to have perceived in order by sequentially selecting from nine alternative letters, which included both targets and distractors in their temporal vicinity, presented in a matrix pattern on the screen. They responded with buttons in the numeric keypad of a standard keyboard spatially mapping the letter matrix and were encouraged to guess when uncertain about the target. Participants in the implicit-timing group passively viewed trial sequences and reported target identities, similar as in the conventional AB task [2]. Participants in the explicit-timing group were additionally prompted to rate the perceived inter-target interval after reporting targets using four alternatives: “only see one target (1)”, “relatively short (2)”, “medium (3)”, and “relatively long (4)”. Once their responses were registered, the next trial was initiated after an 800–1000 ms inter-trial interval.

All participants performed two randomly ordered blocks, each containing 240 trials (Figure 1B). In one block, referred to as the “time-cue present vs. absent” block, both single-target trials (only T1 present) and double-target trials (lag1 and lag3 conditions with both T1 and T2 present) were included. Depending on its preceding trial, a double-target trial could be classified as either a time-cue present trial (e.g., the preceding trial and the current trial were both lag1 or lag3 trials) or a time-cue absent trial (e.g., the preceding trial was a single-target trial and the current trial was a lag1 or lag3 trial). In either half of the block, single-target trials (50%) were randomly intermixed with either double-target lag1 trials (50%) or double-target lag3 trials (50%). We chose the computer-generated random trial sequence to guarantee that each of the above four conditions (i.e., time-cue present lag1 and lag3, time-cue absent lag1 and lag3) had 30 trials. In the other block, referred to as the “time-cue short vs. long” block, all trials were double-target trials. To make it plausible for the time-cue short vs. long comparison, our analysis focused on the lag3 trial and its relationship with the preceding trial. Similar to the above classification, a lag3 trial was considered to correspond to either the time-cue short, time-cue same, or time-cue long condition, if the preceding trial was a lag1, lag3, or lag5 trial, respectively. Trials of different lag conditions followed a ratio of 1:2:1 and were randomly intermixed in the block according to sequences that resulted in about 39 trials (39.4 ± 2.9) for each of the above conditions (i.e., time-cue short, time-cue same, and time-cue long). Participants were allowed to rest for a while when halfway through each block and forced to rest for at least three minutes between blocks.

#### 2.1.3. Data Analysis

We followed the traditional way in the AB task to calculate T1 and T2 accuracies, i.e., T1 accuracy was computed as the percentage of correct identification, whereas T2 accuracy was conditional on T1 being correctly identified. All descriptive data were presented as mean ± SD. We aimed to investigate whether the processing of time cues in the preceding trial ever modulated the target identification in the current trial. In doing so, we first checked the differences between situations with and without preceding time cues, i.e., time-cue present vs. absent. Then, we examined whether and how the length of the preceding interval cue affected the identification of both targets, i.e., time-cue short vs. same vs. long. We also explored whether the explicit processing of interval cues in the preceding trial would modulate the perceived interval between targets in the current trial. To do so, we performed separate mixed ANOVAs on T1 and T2 accuracies, and repeated-measures ANOVAs on rating scores. Our analysis was primarily on lag3 trials, due to its clear demonstration of the AB effect [43]; other sorts of trials were also examined when enough data were available. Significant interactions were further examined using follow-up simple main effects. Multiple comparisons were corrected with the Bonferroni method. Null results were further confirmed with Bayesian statistics when necessary, where BF_01_ > 1 denoted evidence in favor of the null hypothesis [44].

### 2.2. Results and Discussion

#### 2.2.1. T1 Accuracy

**Figure 2:**
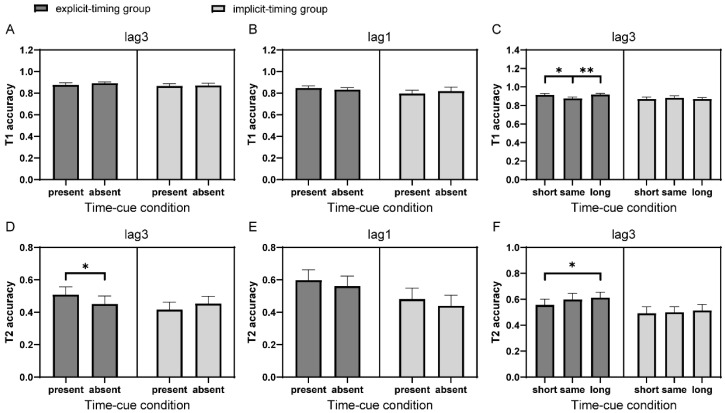
T1 and T2 performances in Experiment 1. (**A**) T1 performances of lag3 trials in the time-cue present vs. absent comparison for participants in the explicit-timing and implicit-timing groups. (**B**) The same as (**A**) of lag1 trials. (**C**) T1 performances of lag3 trials in the time-cue short vs. long comparison for participants in the explicit-timing and implicit-timing groups. (**D**–**F**) The same as (**A**–**C**) for T2 performances. Error bars represent standard error. * *p* < 0.05; ** *p* < 0.01. All multiple comparisons were Bonferroni corrected.

The overall mean accuracy for T1 was 86.3% ± 7.1%. For the time-cue present vs. absent comparison, a 2 (group: explicit-timing vs. implicit-timing; between-subject factor) × 2 (time-cue condition: present vs. absent; within-subject factor) mixed ANOVA analysis was conducted on T1 performance of lag3 trials. No main effects or interactions were found for the group and time-cue condition (Figure 2A). Similar analysis was also performed on lag1 T1 accuracy, and no significant effect was found (Figure 2B).

For the time-cue short vs. long comparison, a 2 (group) × 3 (time-cue condition: short vs. same vs. long; within-subject factor) mixed ANOVA analysis was conducted on T1 performance of lag3 trials. A significant interaction between group and time-cue condition was observed, F(2, 78) = 3.90, *p* = 0.024, η_p_^2^ = 0.09. Simple main effects analysis indicated that T1 performance in the explicit-timing group (F(1.51, 30.26) = 6.42, *p* = 0.004, η_p_^2^ = 0.24), but not in the implicit-timing group (F(2, 38) = 0.38, *p* = 0.69), was modulated by different preceding interval conditions (Figure 2C). In the explicit-timing group, T1 accuracy of lag3 trials was significantly lower when the preceding trial was lag3 than lag1 and lag5 (t(20) = −2.98 and −3.22, p_bonf_ = 0.015 and 0.008, respectively), suggesting that participants with explicit processing of interval information between targets might allocate attentional resources differently with different preceding inter-target intervals.

#### 2.2.2. T2 Accuracy

Consistent with the typical observation in the AB performance [45], the mean accuracy for T2 was much lower (t(40) = −13.02, *p* < 0.001) when the inter-target interval was 300 ms (lag3: 50.4% ± 19.5%) compared with 500 ms (lag5: 75.0% ± 17.3%). The lag1 accuracy (55.7% ± 23.1%), on average, was 5.3% higher than the lag3 accuracy. Although the difference was not significant (t(40) = 1.13, *p* = 0.27), this potential lag1 sparing did not conflict with the literature [46]. Similar to the analysis for T1 accuracy, a 2 (group) × 2 (time-cue condition) mixed ANOVA was performed on T2 accuracy of lag3 trials for the comparison of time-cue present vs. absent. A significant interaction between the group and time-cue condition was found, F(1, 39) = 5.90, *p* = 0.02, η_p_^2^ = 0.13. Follow-up simple main effects analysis showed higher T2 accuracy when the preceding trial was also a double-target (i.e., lag3), compared with a single-target trial only in the explicit-timing group (F(1, 20) = 5.88, *p* = 0.025, η_p_^2^ = 0.23) but not in the implicit-timing group (F(1, 19) = 1.41, *p* = 0.25, BF_01_ = 2.32), suggesting that participants’ performance on T2 could benefit from the explicit processing of valid interval cues in the preceding trials (Figure 2D). Likewise, in lag1 trials, a marginally significant main effect of time-cue condition (F(1, 39) = 3.98, *p* = 0.053, η_p_^2^ = 0.09) revealed a tendency of higher T2 accuracy when the preceding trial was also a lag1 trial (compared with a single-target trial); however, the effect appeared to be independent of whether or not participants paid attention to the temporal information (Figure 2E). The difference between lag3 and lag1 results was likely due to the different number of attentional episodes and nonoverlapping underlying mechanisms [9,46,47]. As we mentioned in the Introduction, we focused our discussion on the AB effect; nevertheless, it is interesting to examine whether and how the temporal information modulates the processes within one attentional episode, thus, the lag1 sparing in future research.

We further compared conditions between time-cue short vs. same vs. long, focusing on lag3 trials with a 2 (group) × 3 (time-cue condition) mixed ANOVA, which showed a marginally significant main effect of time-cue condition (F(2, 78) = 2.54, *p* = 0.085, η_p_^2^ = 0.06). Despite the non-significant interaction, we further compared T2 accuracy in lag3 trials between all pairs derived from time-cue short, time-cue same, and time-cue long conditions separately for the explicit-timing and implicit-timing groups, considering the theoretical implications of such comparisons as suggested in the Introduction and the result of T1 analysis. There was a significant main effect of time-cue condition in the explicit-timing group (F(2, 40) = 4.30, *p* = 0.020, η_p_^2^ = 0.18) but not in the implicit-timing group (F(2, 38) = 0.28, *p* = 0.76). In the explicit-timing group, T2 performance at lag3 was better when the preceding trial was lag5 than lag1 (t(20) = 2.83, p_bonf_ = 0.022), but comparable between lag5 and lag3 preceding time-cue conditions (t(20) = 0.74, p_bonf_ = 1.00, BF_01_ = 3.52; Figure 2F). In contrast, there was no difference between these time-cue conditions in the implicit-timing group (p_bonf_ = 1.00 and 1.00, BF_01_ = 3.30 and 3.88, respectively).

It appears that the interval-related effect of preceding trials could modulate the attentional allocation only when participants purposefully processed the temporal information during the task; thus, the identification of T2, likely also T1, was modulated in the current trial. The requirement of explicitly processing interval information also suggested that participants might gradually learn to exploit the temporal information to aid their attentional allocation. Hence, we further compared T2 accuracies of lag3 trials when the interval cue was present (preceded by a lag3 trial) or long (preceded by a lag5 trial) with those when the interval cue was absent (preceded by a single-target trial) or short (preceded by a lag1 trial), separately for the first and last thirds of trials. Consistent with our hypothesis, the interval cueing effect, i.e., better performance when the interval cue was present/long vs. absent/short, was clearly pronounced in the last third of trials (t(20) = 2.67, *p* = 0.015), but only marginal in the first third of trials (t(20) = 1.81, *p* = 0.085) in the explicit-timing group. In comparison, there was no significant effect in both the first and last thirds of trials in the implicit-timing group (t(19) = −0.02 and 0.61, *p* = 0.99 and 0.55, BF_01_ = 4.30 and 3.64, respectively).

#### 2.2.3. Rating of Perceived Inter-Target Interval

To verify whether participants were sensitive to the time intervals between targets, we inspected the rating scores in different lags, including the single-target condition. It is obvious that with the increase in lags (or the inter-target intervals; the single-target condition could be considered as having an infinitely short interval), the rating scores changed towards relatively longer perceived intervals (single-target: 1.17 ± 0.33, lag1: 1.76 ± 0.38, lag3: 2.43 ± 0.51, and lag5: 2.91 ± 0.70; repeated-measures ANOVA: F(1.87, 37.44) = 64.96, *p* < 0.001, η_p_^2^ = 0.77, all pair-wise ps < 0.005 after Bonferroni correction), confirming that our manipulation of explicit-timing was successful and participants were able to use such time cues.

Next, we examined whether different interval cue conditions might alter the perceived inter-target interval. To do so, we conducted repeated-measures ANOVAs in the time-cue present vs. absent comparison on the rating scores of lag3 and lag1 trials, separately. No significant difference was found between conditions of preceding double-target and single-target trials (F(1, 20) = 0.32 and 1.00, *p* = 0.58 and 0.33, for lag3 and lag1, respectively; Figure 1C). This result is not surprising, considering that the single-target trial did not convey specific inter-target interval information so that no relative length information was available in the block to modulate the perceived interval in the current trial.

More interestingly, we observed a significant main effect of time-cue condition in the time-cue short vs. long comparison where the information of inter-target interval relativity was readily achievable (F(2, 40) = 4.95, *p* = 0.012, η_p_^2^ = 0.20). This effect was primarily manifested by the longer perceived inter-target interval when the current lag3 trial was preceded by a lag5 than lag1 (t(20) = 2.73, p_bonf_ = 0.028) or lag3 (t(20) = 2.73, p_bonf_ = 0.028) trial (Figure 1D). Together with the results of T2 accuracy, this observation implies that when participants explicitly processed a longer inter-target interval, they were better prepared for T2 processing in the following trial. Another possibility, certainly a more provoking and worthy of further investigation one, is that the temporal allocation of resources on T2 depends on the perceived interval between T1 and T2; with longer apparent inter-target intervals (e.g., lag3 following lag5 in the present case), resources are more efficiently allocated to identify T2.

## 3. Experiment 2: Effect of Rhythmic Cues

### 3.1. Method

#### 3.1.1. Participants

Forty-five healthy young adults, 44 from Experiment 1 and one who was newly recruited, participated in Experiment 2 (nine males, 19.87 ± 0.97 years). Like in Experiment 1, the sample size was sufficient according to the power analysis, and participants were randomly assigned into the “explicit-timing” group and the “implicit-timing” group. After excluding participants with an overall T1 accuracy lower than two standard deviations from the group mean, there were 21 participants in the explicit-timing group and 20 in the implicit-timing group. Participants in both groups were not informed of the presentation manipulation before the task. All participants provided informed consent before and received reimbursement after the experiment. This procedure was in accordance with the Declaration of Helsinki and approved by the Ethics Committee in Shanghai University of Sport.

#### 3.1.2. Apparatus, Stimuli, and Procedure

**Figure 3:**
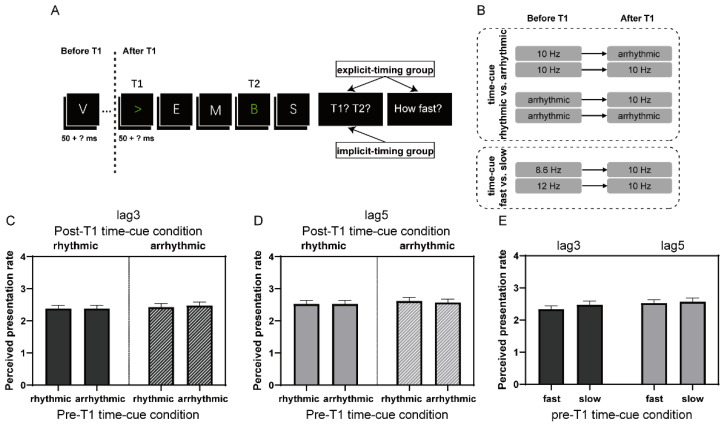
The experimental paradigm of the AB task and subjective rating scores of time perception in Experiment 2. (**A**) Schematic representation of a single trial in Experiment 2. (**B**) The temporal regularities before and after T1 were manipulated separately within trials. (**C**) Ratings of perceived presentation rate in the time-cue rhythmic vs. arrhythmic comparison at lag3. (**D**) The same as (**C**) at lag5. (**E**) Ratings of perceived presentation rate in the time-cue fast vs. slow comparison at lag3 and lag5, respectively. The vertical axis indicates “not clear (1)”, “relatively fast (2)”, “medium (3)”, and “relatively slow (4)”. Error bars represent standard error.

The apparatus and visual stimuli were identical to those in Experiment 1, except that T1 was a symbol (either “<” or “>”; Figure 3A). The change in T1 mainly followed the observation of low T1 accuracy in our pilot experiment (mean: 76.8%; thus, reducing valid trials for calculating T2 accuracy), with T1 being letters and the presentation rates being manipulated. This was probably due to the stream rate-related effects on T1 accuracy [25]. Like in Experiment 1, participants in the implicit-timing group passively viewed the RSVP stream and reported targets afterwards. For participants in the explicit-timing group, they were, in addition, prompted to rate the perceived presentation rate of the stream after reporting targets using four alternatives: “not clear (1)”, “relatively fast (2)”, “medium (3)”, and “relatively slow (4)”.

The procedure was also similar to Experiment 1, except the following (Figure 3B): The temporal regularities of the visual stream before and after T1 were independently manipulated. In two “time-cue rhythmic vs. arrhythmic” blocks, the items either before or after T1 were presented rhythmically (10 Hz) or arrhythmically, thus, generating four time-cue conditions for both lag3 and lag5 trials (39 trials for each lag and time-cue condition): 10 Hz to 10 Hz, arrhythmic to 10 Hz, 10 Hz to arrhythmic, and arrhythmic to arrhythmic. Items with rhythmic presentation were displayed for 50 ms and separated by 50 ms blank inter-stimulus intervals, whereas items with arrhythmic presentation were displayed again for 50 ms but had variable inter-stimulus intervals, whose total duration was kept the same as that of rhythmic presentation. In the “time-cue fast vs. slow” block, the presentation rate before T1 was set at 12 Hz (letter for 50 ms and blank for 33 ms) or 8.6 Hz (letter for 50 ms and blank for 66 ms), while the presentation rate after T1 was fixed at 10 Hz (letter for 50 ms and blank for 50 ms). Two time-cue conditions were generated for both lag3 and lag5 trials in this block (39 for each lag and time-cue condition): 12 Hz to 10 Hz and 8.6 Hz to 10 Hz. To make participants better appreciate the rhythm of presentation, 21–25 items were included in the RSVP and T1 randomly appeared at 1200 ms, 1300 ms, or 1400 ms after the onset of the stream. T2 was always presented at 300 ms and 500 ms following T1, respectively, for the lag3 and lag5 conditions, regardless of the presentation rate. No repetition of letters was permitted from T1 onset to trial-end. All participants performed the three above-described blocks in random order, with 156 trials in each block.

#### 3.1.3. Data Analysis

The calculation of T1 and T2 accuracies was the same as in Experiment 1. In this experiment, we first examined whether a certain rhythm present in the RSVP stream would facilitate the identification of both T1 and T2, i.e., time-cue rhythmic vs. arrhythmic. Then, we proceeded to check whether perceiving the change in the presentation rate played a role in the AB performance, i.e., time-cue fast vs. slow. We also explored whether the explicit processing of rhythmic cues would modulate the perceived presentation rate of the RSVP stream. Like in Experiment 1, we applied mixed ANOVAs on T1 and T2 accuracies, and repeated-measures ANOVAs on rating scores, with the primary interest in lag3 trials but also with an interest in lag5 trials. Significant interactions were further examined using follow-up simple main effects.

### 3.2. Results and Discussion

#### 3.2.1. T1 Accuracy

**Figure 4:**
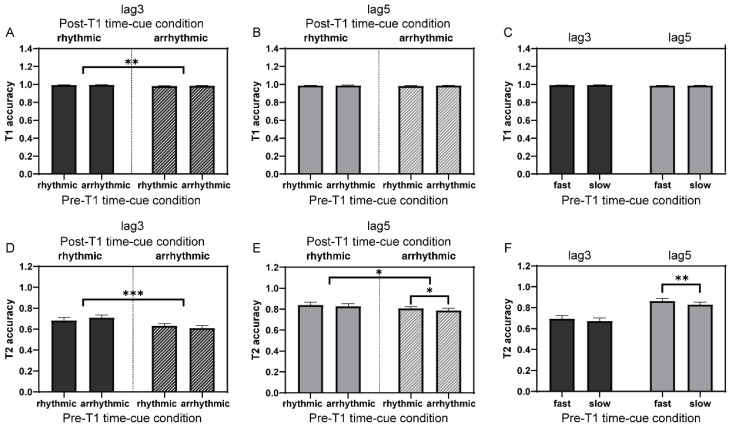
T1 and T2 performances (collapsed across groups) in Experiment 2. (**A**) T1 performances of lag3 trials in the time-cue rhythmic vs. arrhythmic comparison. (**B**) The same as (**A**) of lag5 trials. (**C**) T1 performances of lag3 and lag5 trials in the time-cue fast vs. slow comparison. (**D**–**F**) The same as (**A**–**C**) for T2 performances. Error bars represent standard error. * *p* < 0.05; ** *p* < 0.01; *** *p* < 0.001.

The overall mean accuracy for T1 was 98.7% ± 1.2%. For T1 accuracy of lag3 trials in the time-cue rhythmic vs. arrhythmic comparison, a 2 (group: implicit-timing vs. explicit-timing; between-subject factor) × 2 (pre-T1 time-cue condition: rhythmic vs. arrhythmic; within-subject factor) × 2 (post-T1 time-cue condition: rhythmic vs. arrhythmic; within-subject factor) mixed ANOVA was conducted. There was a significant main effect of post-T1 time-cue condition (F(1, 39) = 10.58, *p* = 0.002, η_p_^2^ = 0.21), suggesting that the presence of the post-T1 rhythmicity promoted T1 performance (Figure 4A). Similar analysis was also performed on T1 accuracy of lag5 trials, and no significant effect was found (Figure 4B).

For trials in the time-cue fast vs. slow comparison, we performed 2 (group) × 2 (pre-T1 time-cue condition: fast vs. slow; within-subject factor) mixed ANOVAs on T1 accuracies of lag3 and lag5 trials, separately. No significant effect was ever observed (Figure 4C).

#### 3.2.2. T2 Accuracy

The mean accuracy for T2 was much lower (t(40) = −9.14, *p* < 0.001) when the inter-target interval was 300 ms (lag3: 66.6% ± 16.2%) compared with 500 ms (lag5: 82.6% ± 13.4%), in accordance with the typical pattern in the AB performance [45]. Similar to the analysis for T1 performance, a 2 (group) × 2 (pre-T1 time-cue condition) × 2 (post-T1 time-cue condition) mixed ANOVA was conducted to analyze T2 performance of lag3 trials in the time-cue rhythmic vs. arrhythmic comparison. The result showed a significant main effect of post-T1 time-cue condition (F(1, 39) = 25.45, *p* < 0.001, η_p_^2^ = 0.40), which was characterized by a better T2 performance when the presentation after T1 was rhythmic than arrhythmic (Figure 4D). There was also a significant interaction between pre-T1 and post-T1 time-cue conditions (F(1, 39) = 5.00, *p* = 0.031, η_p_^2^ = 0.11). Follow-up simple main effects did not reach significance, but only showed a tendency that the arrhythmic presentation of pre-T1 items relatively facilitated T2 performance when the post-T1 frequency was rhythmic (F(1, 39) = 2.91, *p* = 0.096). For T2 accuracy of lag5 trials, similar mixed ANOVA revealed a significant main effect of post-T1 time-cue condition (F(1, 39) = 4.82, *p* = 0.034, η_p_^2^ = 0.11; Figure 4E), which corroborated the observation for lag3 trials and further highlighted the beneficial effect of the post-T1 temporal regularity on T2 identification. For the manipulation of the pre-T1 temporal regularity, a tendency of higher T2 accuracy with the pre-T1 rhythmic compared with arrhythmic presentation was also manifested (F(1, 39) = 4.02, *p* = 0.052, η_p_^2^ = 0.09), showing a potential role of the pre-T1 temporal regularity on T2 performance.

The above results suggested a more important role of the post-T1 temporal regularity in modulating T2 performance. The effect of the pre-T1 temporal regularity, on the other hand, was weakly demonstrated and only showed some tendencies. As the comparison between rhythmic and arrhythmic only reflects one aspect of temporal regularity, other aspects, such as the pace or presentation rate, may also play a role in shaping T2 report. We, thus, compared T2 accuracies between conditions when the frequency before T1 was higher and lower than that after T1, which was fixed at 10 Hz, in the time-cue fast vs. slow comparison. First, T2 accuracy of lag3 trials was submitted to a 2 (group) × 2 (pre-T1 time-cue condition) mixed ANOVA. Neither a significant main effect nor interaction was found, indicating that the pre-T1 stimulus presentation rate had limited, if any, influence on the identification of T2, at least at lag3. When lag5 trials were examined by a similar mixed ANOVA, a significant main effect of pre-T1 time-cue condition was revealed (F(1, 39) = 11.53, *p* = 0.002, η_p_^2^ = 0.23), showing better T2 report if pre-T1 items were displayed at a relatively higher than lower frequency (Figure 4F). Together with the results from the time-cue rhythmic vs. arrhythmic comparison, it seemed that the post-T1 temporal regularity had a general beneficial effect on T2 identification, and also likely on T1 when the competition between targets was intense, whereas the role of the pre-T1 temporal regularity was more variable and dependent on the lag configuration.

#### 3.2.3. Rating of Perceived Presentation Rate

We further explored whether different rhythmic cue conditions might alter participants’ perceived presentation rate of the stream. Therefore, we conducted a 2 (pre-T1 time-cue condition) × 2 (post-T1 time-cue condition) repeated-measures ANOVA on rating scores of lag3 trials for the time-cue rhythmic vs. arrhythmic comparison and a one-way (pre-T1 time-cue condition) repeated-measures ANOVA on rating scores of lag3 trials for the time-cue fast vs. slow comparison. The result revealed neither a significant main effect nor interaction of time-cue condition for both rhythmic vs. arrhythmic and fast vs. slow comparisons (ps > 0.08; Figure 3C,E). A similar negative result was found when performing ANOVAs on rating scores of lag5 trials (ps > 0.3; Figure 3D,E). These results, together, suggested that the processing of subtle differences in the presentation rate in the current experiment most likely operated implicitly. Nevertheless, T2 performance, in some cases also T1 performance, was modulated by the temporal regularity and its alteration during the RSVP stream, especially by that after T1 but also with some evidence by that before T1. Such results were in clear contrast to those observed in Experiment 1, where the effect of interval cues was most obvious when the interval information was explicitly, rather than implicitly, processed.

## 4. General Discussion

The temporal constraint of reporting successive targets in a rapidly presented visual stream is well-known and has attracted a wide range of interest in the research of attentional control and conscious visual perception. The related AB phenomenon is long assumed to be inevitable, but recent studies have revealed ways to attenuate such AB deficit [8,48,49]. Here, we further showed that the immediate temporal experience, based on either T1-T2 intervals or stimulus presentation rates, could modulate performances in an AB task where the target onset time in each trial was unpredictable. Specifically, the T2 accuracy was promoted by the presence of an interval cue (i.e., the lag) in the preceding trial when participants explicitly processed the temporal information, an effect more prominent when the interval was longer. In parallel, the presence of rhythmic cues in the post-T1 stream, independent of whether being explicitly or implicitly processed, generally improved T2 performance, whereas the effect of the pre-T1 temporal regularity was not unanimous and was dependent on the lag configuration. In addition, T1 performances were also modulated in some cases, especially when the competition between targets was intense. Together, our findings indicate that both interval and rhythmic cues, though themselves not reliably predicting the temporal occurrence of targets, can modulate the target identification in the AB task, suggesting a relation between the time processing and resource allocation that is important for conscious visual perception. Considering that the current study did not manipulate stimulus properties or participants’ gross attentional concentration, the results are unlikely explained in terms of central resource limitation or bottleneck [4,5]; rather, they support theories emphasizing the inefficient control of attentional resources—e.g., along the temporal dimension—as the underlying mechanism of the AB phenomenon [6,7,8].

The most obvious observation in our study was that the target identification, especially T2, could be facilitated when the information about temporal structures between T1 and T2 was exploited. When this information was provided by the interval between T1 and T2, it was reminiscent of a foreperiod, which typically refers to the interval between a warning signal and the following target [50] and usually leads to a temporal expectancy according to the hazard function (i.e., the probability of target occurrence) or memory traces [51,52]. In the present study, the temporal expectancy following the processing of T1-T2 interval in the preceding trial likely rendered participants to adopt certain strategies to re-allocate attentional resources in the current trial, such as one attentional episode when preceded by a lag1 trial, two highly overlapped and competing episodes when preceded by a lag3 trial, or two separated episodes when preceded by a lag5 trial. Such adjustment of processes could follow a mere sequential effect [31], without the need of temporal information reliably signaling the occurrence of targets. Indeed, the rating scores of the lag3 inter-target interval differed among situations of preceding trials with different lags, confirming that temporal information could be checked to alter the processes in successive trials. As such, participants’ allocation of attentional resources might also be indirectly modulated by the interval perception in the current trial, a possibility awaiting further examination in future research. Usually, only T2 performances would benefit from such time cues, but in certain cases, such as lag3 trials where T1 and T2 competed strongly for processing resources, the T1 performances also suffered, as shown by our results. One may assume that the confidence following trials with short and long lags might be different. Although this may potentially regulate the performance in the following trial, it is not sufficient to account for the results, given that participants in both explicit-timing and implicit-timing groups had similar confidence levels, but only those purposely processed the interval cues showed the above effect. It is most likely the exploitation of temporal information in the preceding trial that modulated the allocation of resources in the current trial, leading to altered behavioral performance.

When the temporal information was based on the rhythmicity between T1 and T2, the target identification could be enhanced through attentional entrainment [33,53,54]. Particularly, neural entrainment in the alpha band (about 10 Hz) has been demonstrated to modulate visual perception [55] and working memory consolidation [56]. As a result, the rhythmicity between T1 and T2 probably guided attentional resources to on-beat targets and alleviated the competition between T1 and T2, thus, facilitating T2 as well as T1 performances, especially in lag3 trials where the competition was intense. As AB studies typically employed RSVPs with a constant presentation rate, this benefit of rhythmic cues between T1 and T2 might only be revealed when compared with the arrhythmic situation.

We also observed that the information about temporal structures provided by rhythmic cues before T1 modulated T2 performances, which was more pronounced in lag5 trials. We speculated that the rhythmicity and the fast presentation rate before T1 might speed up T1 processing through more temporally efficient allocation of resources on T1 processing due to the attentional entrainment [32,53] and improved temporal segregation between T1 and following distractors [57,58]. The AB effect has been suggested to be associated with an overlap in time between T1-evoked P3 and T2-evoked N2 [59]. Thus, if T1 processing is speeded up, the T2 performance at lag5 may get the advantage of the reduced overlap between T1-evoked P3 and T2-evoked N2. For T2 at lag3, however, little benefit is expected because lag3 is well within the AB window and the temporal separation between T1-evoked P3 and T2-evoked N2 is not large enough to circumvent the overlap (or competition).

By manipulating the temporal regularity or presentation rate, previous studies observed a larger AB effect when stimuli were presented at ~10 Hz compared with arrhythmically or at other frequencies [24,25], different from our main results (Figure 4D). The inconsistency may arise from the different experimental setups. In their studies, different conditions were organized in separate blocks, making it likely that observers were in different temporal strategies or processing states under different conditions; thus, the results reflected more on the inhibitory role of alpha activities in visual perception [60,61]. In our study, conditions were randomly mixed in the same block, and it was likely that the instantaneous temporal information was exploited to mainly facilitate the allocation of attentional resources in the time domain. Hence, previous studies and the current study probably reflect, not exclusively to each other, differently weighted functional aspects of alpha-band neural entrainments [53,60,61]. Moreover, the change in rhythmic presentation within the RSVP stream might also create unknown oscillatory interactions which could not be addressed in the current study, making it more complicated when directly comparing with previous studies. While it is currently difficult to resolve the responsible factors leading to different results and further research is needed, the current study may better reflect the immediate influences of pre-T1 and post-T1 temporal regularities on the target identification in the AB task.

A network involving the frontal and parietal cortices is assumed as the likely neural basis of the AB phenomenon [10,11,59,62]. Part of the same neural circuitry is also implied in the time experience [63], with activities in the parietal and frontal regions exhibiting patterns consistent with the hazard rate [64] and internal timing [65,66]. Subcortical areas, such as the cerebellum and basal ganglia, are also involved in the attentional switching [67], error prediction [68], and event timing [37]. These shared cortical and subcortical structures, thus, may provide a platform to support the interaction between timing and attention [69] which can partially explain our results. Interestingly, the interval-based and rhythm-based timing are known to recruit nonoverlapping neural circuits [36,37] and operate on different levels of conscious control [38,39]. Our results, likewise, showed that the effect of interval cues required participants to explicitly process the temporal information whereas that of rhythmic cues did not, suggesting a further dissociation in mechanisms underlying the interaction between timing and temporal attention which depends on the nature of time cues.

## 5. Conclusions

In conclusion, our findings indicate that observers can exploit the instantaneous temporal information from both interval and rhythmic cues to aid their judgment of successive targets in an RSVP stream, even though these cues do not convey reliable information about when targets will occur. Such cognitive processes reflect the flexibility of deploying attentional resources in the time dimension [32] as well as the potential advantage of adapting to the environmental dynamics. Future studies may proceed to further delineate the effect profile across finely tuned lags and compare the short-term (through immediate experience) and long-term (through statistical learning) time-cueing effects. It is also worthy of examining the interplay between temporal processing and attentional control, and the relevant neural circuits, with some focus on the differences between various time cues (e.g., interval-based and rhythm-based) as well as the respective modulatory effects on T1 and T2 processing.

## Data Availability

Data will be made available from the authors upon request.
